# The anatomy of choice: dopamine and decision-making

**DOI:** 10.1098/rstb.2013.0481

**Published:** 2014-11-05

**Authors:** Karl Friston, Philipp Schwartenbeck, Thomas FitzGerald, Michael Moutoussis, Timothy Behrens, Raymond J. Dolan

**Affiliations:** 1 The Wellcome Trust Centre for Neuroimaging, University College London, 12 Queen Square, London WC1N 3BG, UK; 2 Centre for Functional MRI of the Brain, The John Radcliffe Hospital, Headley Way, Oxford OX3 9DU, UK

**Keywords:** active inference, agency, Bayesian inference, bounded rationality, free energy, utility theory

## Abstract

This paper considers goal-directed decision-making in terms of embodied or active inference. We associate bounded rationality with approximate Bayesian inference that optimizes a free energy bound on model evidence. Several constructs such as expected utility, exploration or novelty bonuses, softmax choice rules and optimism bias emerge as natural consequences of free energy minimization. Previous accounts of active inference have focused on *predictive coding*. In this paper, we consider *variational Bayes* as a scheme that the brain might use for approximate Bayesian inference. This scheme provides formal constraints on the computational anatomy of inference and action, which appear to be remarkably consistent with neuroanatomy. Active inference contextualizes optimal decision theory within embodied inference, where goals become prior beliefs. For example, expected utility theory emerges as a special case of free energy minimization, where the *sensitivity* or inverse temperature (associated with softmax functions and quantal response equilibria) has a unique and Bayes-optimal solution. Crucially, this sensitivity corresponds to the *precision* of beliefs about behaviour. The changes in precision during variational updates are remarkably reminiscent of empirical dopaminergic responses—and they may provide a new perspective on the role of dopamine in assimilating reward prediction errors to optimize decision-making.

## 1. Introduction

This paper considers decision-making and action selection as variational Bayesian inference. It tries to place heuristics in decision theory (in psychology) and expected utility theory (in economics) within the setting of embodied or active inference. In brief, we treat the problem of selecting behavioural sequences or policies as an inference problem. We assume that policies are selected under the prior belief that they minimize the difference (relative entropy) between a probability distribution over states that can be reached and states that agents believe they should occupy. In other words, choices are based upon beliefs about alternative policies, where the most likely policy minimizes the difference between attainable and desired outcomes. By formulating the problem in this way, three important aspects of optimal decision-making emerge.

First, because relative entropy can always be decomposed into entropy and expected utility, the ensuing choices necessarily maximize both expected utility and the entropy over final states. This is closely related to maximizing extrinsic and intrinsic rewards in embodied cognition and artificial intelligence. In this setting, utility or *extrinsic reward* is supplemented with *intrinsic reward* to ensure some efficient information gain, exploratory behaviour or control over outcomes. Important examples here include artificial curiosity [1], empowerment [2], information to go [3], computational complexity [4] and self-organization in non-equilibrium systems [5]. In the current setting, a policy that maximizes the entropy over final states is intrinsically rewarding because it keeps ‘options open’.

Second, because choices are based upon beliefs about policies, these beliefs must be associated with a confidence or precision—that is itself optimized. This furnishes a unique and Bayes-optimal sensitivity or inverse temperature of the sort associated with softmax choice rules and quantal response equilibria (QRE) [6].

Third, because beliefs about policies depend upon beliefs about the current state of the world, and vice versa, there is an inevitable optimism bias [7] in which inferences about ambiguous states are biased towards those that support an optimal policy [8].

We motivate the premises that underlie this formulation and unpack its implications using formal arguments and simulations. These simulations are described in detail in a technical companion paper [8]. The novel contribution of this work is the notion that the brain might use variational Bayes for approximate Bayesian inference—and that this variational scheme provides constraints on the computational anatomy of inference and action. In particular, variational Bayes specifies a unique and optimal precision, where Bayesian updates of expected precision (or confidence about desired outcomes) look very much like dopaminergic responses—providing a new interpretation of dopamine that goes beyond reporting reward prediction errors.

The basic idea behind active inference is that behaviour can be understood in terms of inference: in other words, action and perception are integral parts of the same inferential process and one can only be understood in light of the other. It is fairly straightforward to show that self-organizing systems are necessarily inferential in nature [9]. This notion dates back to Helmholtz and Ashby [10–12] and has been formalized recently as minimizing a variational free energy bound on Bayesian model evidence [13,14]. A corollary of this active inference scheme is that agents must perform some form of *Bayesian inference*. Bayesian inference can be approximate or exact, where exact inference is rendered tractable by making plausible assumptions about the approximate form of probabilistic representations—representations that are used to predict responses to changes in the sensorium. The key question, from this perspective, is how do agents perform approximate Bayesian inference? This contrasts with utilitarian and normative accounts of behaviour, which ask how agents maximize some expected value or utility function of their states [15–17].

Normative approaches assume that *perfectly rational* agents maximize value [18], without considering the cost of optimizing behaviour [19]. By contrast, *bounded rational* agents consider processing costs and do not necessarily choose the most valuable option [20]. Most attempts to formalize bounded rationality focus on the Boltzmann distribution, where optimal behaviour involves choosing states with a high value or low energy [4,21]. For example, QRE models assume that choice probabilities are prescribed by a Boltzmann distribution and that rationality is determined by a *temperature* parameter [6,22]. Related stochastic choice rules have a long history in psychology and economics, particularly in the form of logit choice models [23,24]. These choice rules are known as *softmax rules* and are used to describe stochastic sampling of actions, particularly in the context of the exploration–exploitation dilemma [25,26]. In this setting, the temperature models the *sensitivity* of stochastic choices to value, where perfect rationality corresponds to a very high sensitivity (low temperature). The purpose of this paper is to suggest that sensitivity can itself be optimized and corresponds to the confidence or precision associated with beliefs about the consequences of choices.

In active inference, there is no value function—free energy is the only quantity that is optimized. In this context, bounded rationality is an emergent feature of free energy minimization and the value of a state is a consequence of behaviour producing that state, not its cause. In other words, the consequences of minimizing free energy are that some states are occupied more frequently than others—and these states are valuable. Crucially, in active inference, parameters like sensitivity or inverse temperature must themselves minimize free energy. This means that sensitivity ceases to be a free parameter that is adjusted to describe observed behaviour and becomes diagnostic of the underlying (approximate) Bayesian inference scheme. We will see that sensitivity corresponds to the *precision* of beliefs about the future and behaves in a way that is remarkably similar to the firing of dopaminergic cells in the brain. Furthermore, QRE, logit choice models and softmax rules can be derived as formal consequences of free energy minimization, using variational Bayes.

Variational Bayes or ensemble learning is a general and widely used scheme for approximate Bayesian inference [27]. It rests on a partition of probabilistic representations (approximate posterior probability distributions) that renders Bayesian inference tractable. A simple example would be estimating the mean and precision (inverse variance) of some data, under the approximating assumption that uncertainty about the mean does not depend upon uncertainty about the variance and vice versa. This enables a straightforward computation of descriptive statistics that would otherwise be extremely difficult (see [28] for details). Neurobiologically, a partition into conditionally independent representations is nothing more than functional segregation—in which specialized neuronal systems can be regarded as performing variational Bayesian updates by passing messages to each other. This paper tries to relate variational Bayes to the functional anatomy of inference and action selection in the brain. This provides a functional account of neuronal representations and functional integration (message passing) among different systems. A particularly important example will be the exchange of signals among systems encoding posterior beliefs about precision with systems representing hidden states of the world and action, respectively—an exchange we associate with the convergent control of dopaminergic firing and its divergent influence on Bayesian updates in the prefrontal cortex and striatum.

Although variational Bayes uses discrete updates, variational updates still possess a dynamics that can be compared to neuronal responses, particularly dopaminergic responses. We focus on this comparison because understanding the computational role of dopamine is important for understanding the psychopathology and pathophysiology of conditions such as Parkinson's disease, schizophrenia and autism. Traditionally, dopamine has been associated with the reporting of reward prediction errors [29]. However, this may provide an incomplete account of dopamine, because it fails to account for its putative role in action (e.g. the bradykinesia of Parkinson's disease) and perception (e.g. hallucinations and delusions in schizophrenia). Much of current thinking in computational psychiatry points to dopamine as mediating a representation of uncertainty or precision that can account for both false inference [30–33] and impoverished action [34]. In what follows, we will see how precision relates to value and thereby resolves the dialectic between the role of dopamine in reporting reward prediction errors and as a neuromodulator of action and attentional selection [35,36].

This paper comprises three sections: §2 introduces active inference and describes a general model of control or agency, in which purposeful behaviour rests on prior beliefs that agents will minimize the (relative) entropy of their final states. This leads naturally to expected utility theory and exploration bonuses. §3 considers the inversion of the generative model using variational Bayes, with a special focus on belief updates and message passing. §4 considers the implications for the functional anatomy of inference and decision-making, namely reciprocal message passing between systems supporting perceptual inference, action selection and the encoding of uncertainty or precision.

## 2. Active inference

This section introduces active inference, in which beliefs about (hidden or fictive) states of the world maximize model evidence or the marginal likelihood of observations. In contrast to classic formulations, active inference makes a distinction between *action* that is a physical state of the real world and beliefs about action that we will refer to as *control* states. This changes the problem fundamentally from selecting an optimal action to making optimal inference about control. In other words, under the assumption that action is sampled from posterior beliefs about control, we can treat decision-making and action selection as a pure inference problem that necessarily entails optimizing beliefs about behaviour and its consequences. Sampling actions from posterior beliefs is known as Thompson sampling [37,38]; see [38] which is especially relevant as it provides a free energy derivation.

The following summarizes the material in ref. [8]. We use bold-italic typeface to indicate true states of the world and italic typeface for hidden or fictive states assumed by an agent. The parameters (expectations) of categorical distributions over discrete states 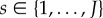 are denoted by *J* × 1 vectors 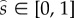, while the ∼ notation denotes sequences of variables over time.

**Definition.**

Active inference rests on the tuple 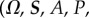 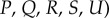

— A finite set of observations *Ω*.
— A finite set of true states and actions ***S*** × *A*.
— A finite set of fictive or hidden states *S* × *U*.
— A *generative process* over observations, states and action 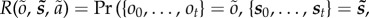 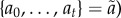.
— A *generative model* over observations and hidden states 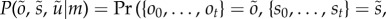 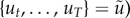.
— An *approximate posterior probability* over hidden states with expectations 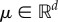 such that 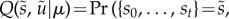 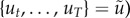.

**Remarks**. In this set-up, the *generative process* describes transitions among real states of the world that depend upon action and generate outcomes. This process models the environment that the agent samples through action. Actions are sampled from approximate posterior beliefs based on a *model* of the generative process. In the generative model, actions *A* are replaced by control states *U*. The generative model is embodied by an agent (denoted by *m*) that is coupled to the environment through observations (sampled from the generative process) and actions (sampled from its posterior beliefs). Finally, approximate posterior beliefs about hidden states *S* × *U* are encoded by expectations 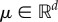.

As it stands, this definition does not describe a process. This is because the dependencies among real states and expectations are not specified. In other words, the agent's generative model of observations 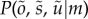 and its approximate posterior distribution over their causes 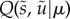 does not refer to the process of eliciting outcomes through action 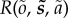. To couple the agent to its environment, we have to specify how its expectations depend upon observations and how its action depends upon expectations. In active inference, the expectations minimize free energy and the ensuing beliefs about control states prescribe action
2.1
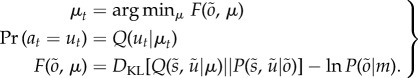



In summary, the environment is characterized as a distribution 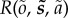 over observations, true states and action, whereas the agent is characterized by two distributions: a generative model 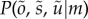 that connects observations to hidden states and posterior beliefs about those states 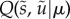 parametrized by its expectations. True states control environmental responses but are never observed directly. Instead, the agent infers hidden states based on its observations. Crucially, these hidden states include control states that prescribe action. Here, the generative model plays a dual role—it is a predictive model over observations and encodes optimal policies (in terms of prior beliefs about control states). The agent and the environment interact in cycles. In each cycle, the agent first figures out which hidden states are most likely by optimizing its expectations with respect to the free energy of observations. After optimizing its posterior beliefs, an action is sampled from the posterior marginal over control states. The environment then picks up this action, generates a new observation and a new cycle starts.

The optimization above is that usually portrayed in terms of *perception* (inference about hidden states) and *action* (a choice model in which action is a function of inferred states). Action and perception couple the agent to the environment; where expectations depend upon observations—through perception, whereas observations depend upon expectations—through action. Usually, expectations are associated with neuronal activity or connection strengths and action is associated with the state of effectors. In brief, expectations about the state of the world minimize free energy, while action is selected from the ensuing posterior beliefs about control states.

The expression for free energy above shows that it upper bounds the negative logarithm of Bayesian model evidence 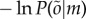 or *surprise*. This is because the relative entropy or Kullback–Leibler (KL) divergence term cannot be less than zero [27]. This means minimizing free energy corresponds to minimizing the divergence between the approximate and true posterior. This formalizes the notion of unconscious inference in perception [10,39,13] and, under some simplifying assumptions, reduces to predictive coding [40].

In summary, minimizing free energy corresponds to approximate Bayesian inference and, in active inference, choosing the least surprising outcomes. However, if agents model their environment, they have to entertain posterior beliefs about the control of state transitions producing outcomes. This means that we have moved beyond classical formulations—in which deterministic actions are selected—and have to consider posterior beliefs about putative choices. In §2*a*, we consider the optimization of posterior beliefs and the confidence or precision with which these beliefs are held.

### (a) A generative model of goal-directed agency

Surprise or model evidence is an attribute of a generative model. This model comprises prior beliefs that determine the states an agent frequents. It is these beliefs that specify the attracting states (goals) that action will seek out—to avoid surprise. We now consider how prior beliefs can be understood in terms of expected utility.

**Figure 1.**
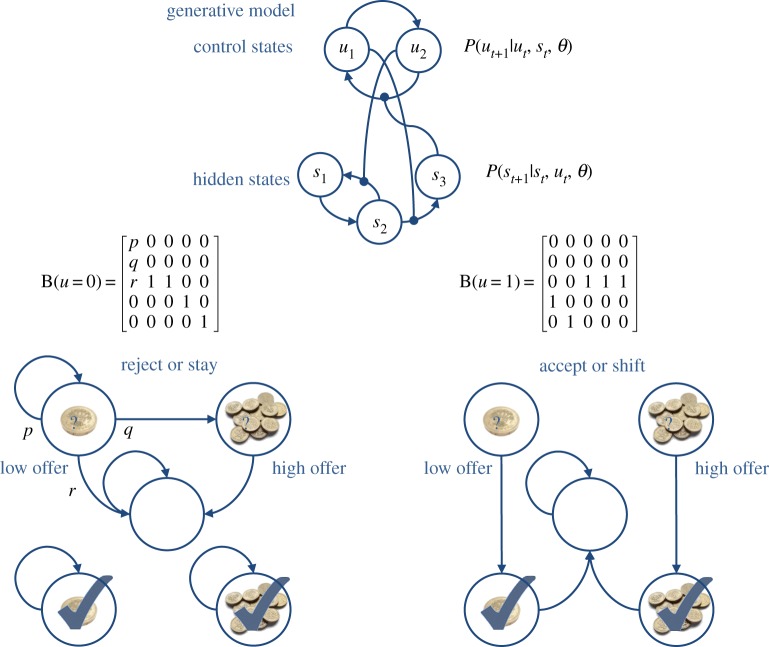
*Upper panel*: a schematic of a hierarchical generative model with discrete states. The key feature of this model is that it entertains a subset of hidden states called control states. The transitions among one subset depend upon the state occupied in the other. *Lower panels*: this provides an example of a particular model with two control states; reject (stay) or accept (shift). The control state determines transitions among hidden states that comprise a low offer (first state), a high offer (second state), a no-offer state (third state) and absorbing states that are entered whenever a low (fourth state) or high (fifth state) offer is accepted. The probability of moving from one state to another is unity, unless otherwise specified by the transition probabilities shown in the middle row. The (hazard rate) parameter *r* controls the rate of offer withdrawal. Note that absorbing states—that re-enter themselves with unit probability—render this Markovian process irreversible. We will use this example in later simulations of choice behaviour.

The models we consider rest on transitions among hidden states that are coupled to transitions among control states. This coupling is illustrated in the upper panel of figure 1. Here, control states modify the transition probabilities among hidden states, while hidden states modify the transitions among control states (as denoted by the connections ending with circles). This form of model allows context-sensitive-state transitions among states generating outcomes—that themselves can induce changes in the control states providing that context. The lower panels of figure 1 depict a particular example that we will use later.

The generative model used to model these (finite horizon Markovian) processes can be expressed in terms of the following likelihood and prior distributions over observations and hidden states to time 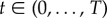 and subsequent control states (omitting normalization constants)
2.2
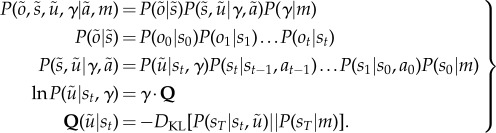



The first equality expresses the generative model in terms of the likelihood of observations given the hidden states (first term), while subsequent terms represent *empirical* prior beliefs. Empirical priors are just probability distributions over unknown variables that depend on other unknown variables. The likelihood says that observations depend on, and only on, concurrent hidden states. The third equality expresses beliefs about state transitions that embody Markovian dependencies among successive hidden states. For simplicity, we have assumed that the agent knows its past actions by observing them. The important part of this generative model lies in the last equalities—describing prior beliefs about control sequences or *policies* that determine which action is selected next.

These beliefs take the form of a Boltzmann distribution, where the policy with the largest prior probability minimizes the relative entropy or divergence between the distribution over final states—given the current state and policy—and the marginal distribution over final states. This marginal distribution defines the agent's *goals* in terms of (desired) states the agent believes it should end up in. One can interpret the negative divergence 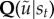 as the *value* of policies available from the current state. In other words, a valuable policy minimizes divergence between expected and desired states. We use 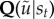 in analogy with action-value in Q-learning [41].

Crucially, the precision of beliefs about policies is determined by a hidden variable 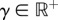 that has to be inferred. We will see in §3 that the expected precision minimizes variational free energy in exactly the same way as expectations about hidden states. There is nothing mysterious about this: we estimate the precision of estimators—by minimizing variational free energy or marginal likelihood—in everyday data analysis when estimating their standard error. In our setting, the expected precision reflects the confidence that goals can be reached from current state. In other words, it encodes the confidence placed in beliefs about optimal outcomes, given observations to date. Note that expected precision is context sensitive and, unlike classical sensitivity parameters, changes with each observation. In summary, this model represents past hidden states and future choices, under the belief that controlled transitions from the current state will minimize the divergence between the distribution over final states and desired states.

### (b) Prior beliefs, entropy and expected utility

Basing beliefs about choices on relative entropy is formally related to KL optimization; particularly, risk sensitive control (e.g. [42]). This is also a cornerstone of utility-based free energy treatments of bounded rationality [4,21]. These schemes consider optimal agents to minimize the KL divergence between controlled and desired outcomes. All we have done here is to equip agents with prior beliefs that they are KL optimal. These beliefs are then enacted through active inference. The advantage of doing this is that the precision of beliefs about control (i.e. sensitivity to value) can now be optimized—because we have cast optimal control as an inference problem. These arguments may seem a bit abstract but, happily, familiar notions like exploration, exploitation and expected utility emerge as straightforward consequences.

The KL divergence can be thought of as a prediction error—not between expected and observed outcomes—but between the final outcomes predicted with and without considering the current state. In other words, the difference between what can be attained from the current state and the goals encoded by prior beliefs. Unlike classic reward prediction errors, this prediction error is a divergence between probability distributions over states, as opposed to a scalar function of states. Value is the complement of this divergence, which means that the value of the current state decreases when a previously predicted reward can no longer be reached from the current state.

Mathematically, value can be decomposed into two terms that have an important interpretation
2.3
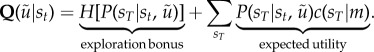



The first is the entropy (intrinsic reward) of the distribution over final states, given the current state and policy. The second is the expected *utility* of the final state, where utility (extrinsic reward) or negative cost is the log probability of the final state under the priors encoding goals: 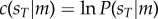.

This decomposition means that agents (believe they) will maximize the entropy of their final states while, at the same time, maximizing expected utility. The relative contribution of entropy and expected utility depends upon the relative utility of different states. If prior goals are very precise (informative), they will dominate and the agent will (believe it will) maximize expected utility. Conversely, with imprecise (flat) priors—that all final states are equally likely—the agent will keep its options open and maximize the entropy over those states: in other words, it will explore, according to the maximum entropy principle [43]. This provides a simple account of *exploration–exploitation* that is consistent with expected utility theory. The entropy term implies that (beliefs about) choices are driven not just to maximize expected value but to explore options in a way that confers an exploratory aspect on behaviour. In the absence of (or change in) beliefs about ultimate states, there will be a bias towards visiting all (low cost) states with equal probability. Similarly, the *novelty bonus* [44] of a new state is, in this formulation, conferred by the opportunity to access states that were previously unavailable—thereby increasing the entropy over final states. This means that the value of a choice comprises an exploration bonus and an expected utility, where the former depends upon the current state and the latter does not.

In summary, if agents occupy a limited set of attracting states, their generative models must be equipped with prior beliefs that controlled state transitions will minimize the divergence between a distribution over attainable states and a distribution that specifies states as attractive. These prior beliefs can be expressed in terms of a KL divergence that defines the value of policies. This value is the same objective function in KL control schemes that grandfather conventional utility-based schemes [4,45]. The value of a policy can be decomposed into its expected utility and an exploration or novelty bonus that corresponds to the entropy over final states. In this setting, notions like value, expected utility and exploration bonus are consequences of the underlying imperative to minimize (relative) entropy. The balance between exploration (entropy) and exploitation (expected value) is uniquely determined by the relative utility of future states—not by inverse temperature: the sensitivity or precision applies to both exploratory and utilitarian behaviour. In other words, explorative behaviour is not just a random version of exploitative behaviour but can itself be very precise, with a clearly defined objective (to maximize the entropy of final outcomes). We will see in §4c that precision plays a different and fundamental role in moderating an *optimism bias* when forming beliefs about hidden states of the world [7]. First, we need to consider the form of the generative model and its inversion.

## 3. Variational bayesian inversion

This section illustrates active inference using the generative model in §2 and its variational Bayesian inversion. To simplify notation, we represent allowable policies with 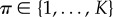, where each policy prescribes a sequence of control states 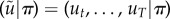. The model considered here is parametrized as follows (omitting constants):
3.1
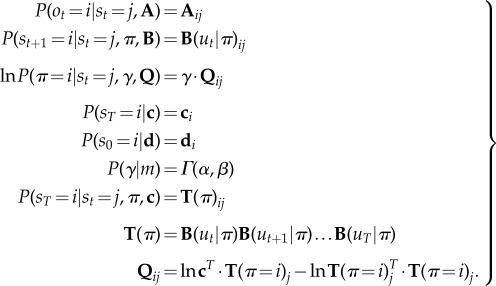



The categorical distributions over observations, given the hidden states, are parametrized by the matrix **A** that maps from hidden states to outcomes. Similarly, the transition matrices 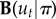 encode transition probabilities from one state to the next under the current policy. The vectors **c** and **d** encode the prior distribution over the last and first states, respectively. The former specify utility 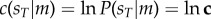. The prior over precision has a standard *γ*-distribution with shape and rate parameters (in this paper) *α* = 8 and *β* = 1. The matrix **Q** contains the values of the *i*th policy from the *j*th hidden state and **T**(*π*) encodes the probability of transition from the current state to a final state, under a particular policy. This is simply the iterated composition of the appropriate transition matrices from the present time until the end of the game.

### (a) Approximate Bayesian inference

Having specified the generative model, we now need to find the expectations that minimize free energy. Variational Bayes provides a generic scheme for approximate Bayesian inference that finesses the combinatoric and analytic intractability of exact inference [27,46]. Variational Bayes rests on a factorization of approximate posterior beliefs that greatly reduces the number of expectations required to encode it. The particular factorization we focus on exploits the Markovian nature of the generative model and has the following form (see [8] for details).
3.2
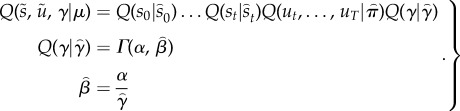



This assumes a factorization over (past) hidden states, (future) control states and precision. The details of the mean field assumption above are not terribly important. The main point is that the formalism of variational Bayes allows one to specify constraints on the form of the approximate posterior that makes prior assumptions or beliefs about choices explicit. For example, in ref. [47], we used a mean field assumption where every choice could be made at every time point. Equation (3.2) assumes that the approximate marginal over precision is, like its conjugate prior, a *γ*-distribution—where the rate parameter is optimized. This rate parameter 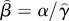 corresponds to temperature in classic formulations. However, it is no longer a free parameter but a sufficient statistic of the unknown precision of beliefs about policies.

Given the generative model in equation (3.1) and the mean field assumption in equation (3.2), the expectations can be expressed as functions of themselves [8] to produce the following remarkably simple variational updates, where 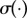 is a softmax function
3.3
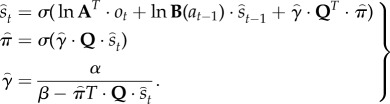



By iterating these equalities until convergence, one obtains a solution that minimizes free energy and provides Bayesian estimates of the hidden variables. This means the expectations change over two timescales—a fast timescale that updates posterior beliefs given the current observations—and a slow timescale that updates posterior beliefs as new observations arrive and action is taken. We have speculated [47] that these updates may be related to nested electrophysiological oscillations, such as phase coupling between *γ*- and *θ*-oscillations in prefrontal–hippocampal interactions [48]. This speaks to biological implementations of variational Bayes, which we now consider in terms of neuronal and cognitive processing.

## 4. The functional anatomy of decision-making

**Figure 2.**
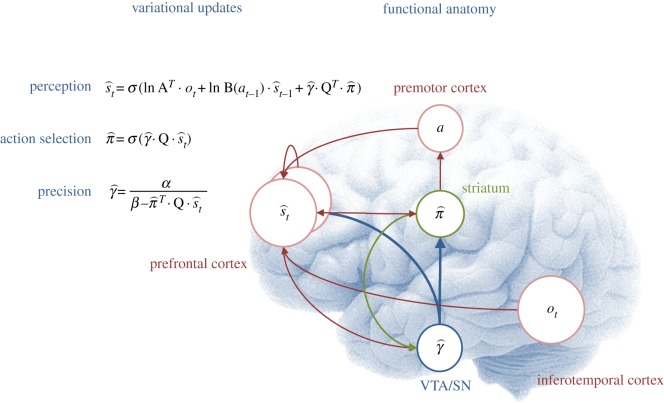
This figure illustrates the cognitive and functional anatomy implied by the variational scheme—or more precisely, the mean field assumption implicit in variational updates. Here, we have associated the variational updates of expected states with perception, of future control states (policies) with action selection and, finally, expected precision with evaluation. The updates suggest the expectations from each subset are passed among each other until convergence to an internally consistent (Bayes optimal) solution. In terms of neuronal implementation, this might be likened to the exchange of neuronal signals via extrinsic connections among functionally specialized brain systems. In this (purely iconic) schematic, we have associated perception (inference about the current state of the world) with the prefrontal cortex, while assigning action selection to the basal ganglia. Crucially, precision has been associated with dopaminergic projections from VTA and SN. See main text for a full description of the equations.

The computational form of variational Bayes resembles many aspects of neuronal processing in the brain: if we assume that neuronal activity encodes expectations, then the variational update scheme could provide a metaphor for *functional segregation*—the segregation of representations, and *functional integration*—the recursive (reciprocal) exchange of expectations during approximate Bayesian inference. In terms of the updates themselves, the expectations of hidden states and policies are softmax functions of (mixtures of) the other expectations. This is remarkable because these updates are derived from basic variational principles and yet have exactly the form of neural networks that use, integrate and fire neurons. Furthermore, the softmax functions are of linear mixtures of expectations (neuronal activity) with one key exception—the modulation by precision when updating beliefs about the current state and selecting the next action. It is tempting to equate this modulation with the neuromodulation by dopaminergic systems that send projections to (prefrontal) systems involved in working memory [49,50] and striatal systems involved in action selection [51,52]. We now consider the variational updates from a cognitive and neuroanatomical perspective (see figure 2 for a summary):

### (a) Perception

The first updates beliefs about the state of the world using observations and beliefs about the preceding state and action. However, there is a third term based upon the expected value of each state, averaged over policies. This can be regarded as an optimism bias in the sense that it biases perception towards high value states—much like dopamine [7]. Figure 2 ascribes these updates to the frontal cortex—assuming neuronal populations here encode the current state. Figure 2 should not be taken too seriously: representations of the current state could have been placed in working memory circuits in the dorsolateral prefrontal cortex [53], ventromedial prefrontal cortex or the anterior cingulate cortex, depending upon the task at hand (e.g. [54]).

### (b) Action selection

The second variational update is a softmax function of the expected value of competing choices under the current state. Figure 2 places this update in the striatum, where the expected value of a policy requires posterior beliefs about the current state from prefrontal cortex and expected precision from the ventral tegmental area (VTA). Crucially, this is the softmax choice rule that predominates in QRE and other normative models [22]. Again, it is remarkable that this utilitarian rule is mandated by the form of variational updates. However, utilitarian theories overlook the symmetry between the expected value over states—that provides the value of a choice, and the expected value over choices—that provides the value of a state. In other words, there are two expected values, one for action 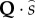 and one for perception 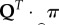. Finally, the expected value under choices *and* states 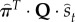 specifies the optimal precision or inverse temperature. Neurobiologically, the softmax policy updates would correspond to biased competition among choices, where precision modulates the selection of competing policies (c.f. [35,36,55]).

### (c) Evaluating confidence

The final variational step estimates the precision of beliefs about policies, using expectations about hidden states and choices. We have associated expected precision with dopaminergic projections from the VTA (and substantia nigra (SN)), which receive messages from the prefrontal cortex and striatum.

The basic tenet of this scheme is that precision must be optimized. So what would happen if (estimated) precision was too high or low? If precision was zero, then perception would be unbiased and represent a veridical representation of worldly states. However, there would be a failure of action selection, in that the value of all choices would be identical. One might heuristically associate this with the pathophysiology of Parkinson's disease—that involves a loss of dopaminergic cells and a poverty of action selection. Conversely, if precision was too high, there would be a predisposition to false perceptual inference—through an augmented optimism bias. This might be a metaphor for the positive symptoms of schizophrenia, putatively associated with hyper-dopaminergic states [31]. In short, there is an optimal precision for any context and the expected precision has to be evaluated carefully on the basis of current beliefs about the state of the world.

In summary, increasing precision biases perceptual inference towards those states that are consistent with prior beliefs about future (choice-dependent) outcomes and increases the precision of action selection. Crucially, the update for expected precision is an increasing function of value, expected under current beliefs about states and choices. This means that the optimal precision depends upon the attainability of goals: if a goal cannot be obtained from the current state, then precision will be low—reducing confidence in predictions about behaviour. Conversely, if there is a clear and precise path from the current state to a goal, then precision will be high. In short, precision encodes the confidence that a goal can be attained and reports the expected value—it plays a dual role in biasing perceptual inference and action selection. We will now look more closely at the neurobiology of precision and can consider not just the role of precision but also how it is controlled by the representations (posterior expectations) it optimizes.

### (d) Precision, dopamine and decision-making under uncertainty

**Figure 3.**
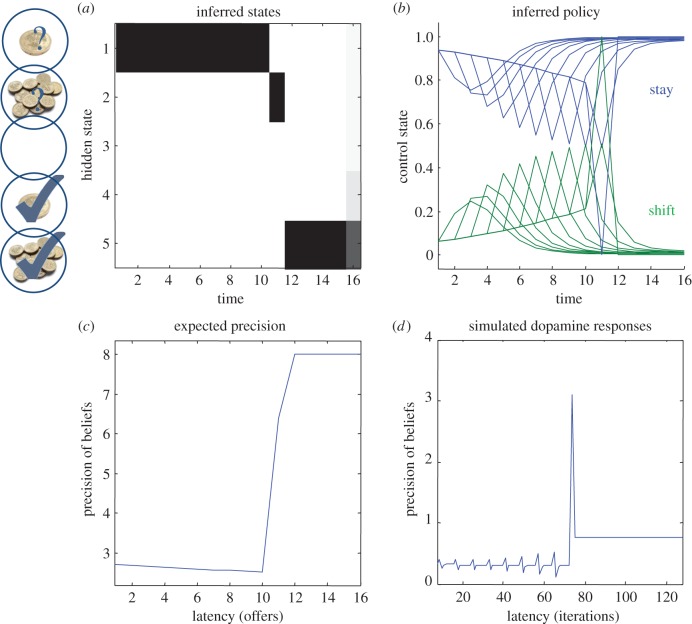
This figure shows the results of a simulation of 16 trials, where a low offer was replaced by high offer on the 11th trial, which was accepted on the subsequent trial. Panel (*a*) shows the expected states as a function of trials or time, where the states are defined in figure 1. Panel (*b*) shows the corresponding expectations about control in the future, where the dotted lines are expectations during earlier trials and the full lines correspond to expectations during the final trial. Black corresponds to reject (stay) and grey to accept (shift). Panels (*c*,*d*) show the time-dependent changes in expected precision, after convergence on each trial (*c*) and deconvolved updates after each iteration of the variational updates (*d*).

Figure 3 shows a simulation based on the transition probabilities in figure 1 (see [8] for details). In this ‘limited offer’ game, the agent has to choose between a low offer—that might be withdrawn at any time—and a high offer—that may replace the low offer with some fixed probability. The problem the agent has to solve is how long to wait. If it waits too long, the low offer may be withdrawn and it will end up with nothing. Conversely, if it chooses too soon, it may miss the opportunity to accept a high offer. In this example, the low offer was replaced with a high offer on the eleventh trial, which the agent accepted. It accepts because this is most probable choice, under its prior belief that it will have accepted the higher offer by the end of the game. The expected probabilities of staying or shifting are shown in the upper right panel (in blue and green, respectively), as a function of time for each trial (thin lines) and the final beliefs (thick lines). The interesting thing here is that before the high offer, the agent believes that it will accept the low offer three or four trials in the future. Furthermore, the propensity to accept (in the future) increases with time (see dotted lines). This means that it waits, patiently, because it thinks it is more likely to accept an offer in the future than to accept the current offer.

The expected precision of these posterior beliefs is shown in the lower left panel and declines gently until the high offer is made. At this point, the expected precision increases markedly, and then remains high. This reflects the fact that the final outcome is assured with a high degree of confidence. These precisions are the expected precisions after convergence of the variational iterations. The equivalent dynamics in the lower right panel show the expected precision over all updates in terms of simulated dopamine responses. These are a least-squares deconvolution of the variational updates, using an exponentially decaying kernel. In other words, these (simulated) dopamine responses reproduce the fluctuations in expected precision when convolved with an exponential kernel (with a time constant of eight iterations). This accounts for the postsynaptic effects of dopamine that, we imagine, decay after its release. The resulting updates show phasic responses to the arrival of new sensory information that converge to tonic values, which minimize free energy.

Many readers will have noted a similarity between the dynamics of precision and the firing of dopaminergic cells. In fact, nearly every anatomical and physiological feature of dopaminergic neurotransmission can be found in these precision updates:

— expected precision modulates the contribution of expected value during the optimization of posterior beliefs about the state of the world and action selection. This fits comfortably with the broadcasting of dopaminergic signals from the VTA and SN to the cortex (for perception) by the mesocortical system—and to the ventral striatum (for action) via nigrostriatal projections. Crucially, the mean field effects implicit in variational Bayes mandate this bilateral (perception and action) dissemination of precision (dopaminergic) signals;
— precision is updated by posterior expectations from the representations it modulates. This is consistent with the projections that control dopaminergic firing in the VTA/SN that are reciprocated by the targets of the ascending dopaminergic system. In other words, nearly every system receiving projections from the VTA projects back to it [56]. Similarly, the main input to the pars reticulata of the SN derives from medium spiny cells in the striatum via the *direct* and *indirect* pathways. These pathways originate in tightly intermingled striatal cells that express different dopamine receptors [57];
— the effect of precision is to modulate the effect of posterior expectations about the current state on control and vice versa. This modulation is exactly congruent with the postsynaptic effects of dopamine: at a synaptic level, dopamine activates G-protein-coupled receptors to modulate the cAMP second messenger system and modify the sensitivity of postsynaptic responses to presynaptic inputs;
— the modulatory role of (expected) precision effectively increases signal to noise during the competition among posterior beliefs about the state of the world (implicit in the softmax function), while doing the same for posterior beliefs about policies. Similarly, dopamine has a dual role in modulating prefrontal cortical responses in working memory circuits [58,53], while at the same time playing a key role in action selection [35,36]. This dialectic may also be reflected by the role of dopamine in schizophrenia and Parkinson's disease [33];
— precision increases monotonically with expected value, where value is composed of an exploration bonus and expected value. Similarly, dopamine is traditionally thought to report novelty, particularly in relation to action [59] and expected value in the same setting [60];
— precision shows phasic (variational update) dynamics in response to new sensory information, which converge to the expected precision. Similarly, dopamine shows characteristic phasic responses to sensory cues that predict rewards, which return to tonic firing levels that may encode uncertainty or predictability [60,61];
— precision increases whenever a predictable path to a goal is signified by sensory input. For example, the appearance of a high offer in figure 3 elicits a greater increase in precision than receipt of the offer *per se*—or its subsequent retention. Similarly, dopamine responses are elicited by sensory cues that, in higher order operant conditioning paradigms, lead to reward but thereafter ‘remain uninfluenced by events that are as good as predicted’ [62]. Indeed, it was the transfer of dopamine responses—from early to late conditioned stimuli—that motivated normative theories of reinforcement learning based upon temporal difference models [29]; and
— precision decreases with the withdrawal of an opportunity to fulfil prior beliefs (shown in [8]). Similarly, dopamine firing decreases in the absence of an expected reward [62].

For people familiar with discussions of dopamine in the context of active inference, the correspondence between precision and dopaminergic neurotransmission will come as no surprise—exactly the same conclusions have been reached when examining predictive coding schemes [34] and hierarchical inference using volatility models [63]. ‘In brief, the emergent role of dopamine is to report the precision or salience of perceptual cues that portend a predictable sequence of sensorimotor events. In this sense, it mediates the affordance of cues that elicit motor behaviour; in much the same way that attention mediates the salience of cues in the perceptual domain.’ [34, p. 1].

## 5. Conclusion

The arguments in this paper can be summarized as follows:

— optimal behaviour can be cast as a pure inference problem, in which valuable outcomes are defined in terms of prior beliefs about future states;
— exact Bayesian inference (perfect rationality) cannot be realized physically, which means that optimal behaviour rests on approximate Bayesian inference (bounded rationality);
— variational free energy provides a bound on Bayesian model evidence (marginal likelihood) that is optimized by bounded rational behaviour;
— bounded rational behaviour requires (approximate Bayesian) inference on both hidden states of the world and (future) control states. This mandates beliefs about action (control) that are distinct from action *per se*—beliefs that entail a precision;
— these beliefs can be cast in terms of minimizing the relative entropy or divergence between prior beliefs about goals and posterior beliefs, given the current state of the world and future choices;
— value can be equated with negative divergence and comprises entropy (exploration or novelty bonus) and expected utility (utilitarian) terms that account for exploratory and exploitative behaviour, respectively;
— variational Bayes provides a formal account of how posterior expectations about hidden states of the world, control states and precision depend upon each other; and may provide a metaphor for message passing in the brain;
— beliefs about the state of the world depend upon expected value over choices, whereas beliefs about choices depend upon expected value over states. Beliefs about precision depend upon expected value under both states and choices;
— precision has to be optimized to balance prior beliefs about choices and sensory evidence for hidden states. In other words, precision nuances an inherent optimism bias when inferring the current state of the world;
— variational Bayes induces distinct probabilistic representations (functional segregation) of hidden states, control states and precision—and highlights the role of reciprocal message passing. This may be particularly important for expected precision that is required for optimal inference about hidden states (perception) and control states (action selection); and
— the dynamics of precision updates, and their computational architecture, are consistent with the physiology and anatomy of the dopaminergic system—providing an account of (mesocortical) projections that encode the precision of valuable states—and (nigrostriatal) projections that encode the precision of valuable actions.

One might ask why these conclusions do not follow from normative accounts of optimal behaviour. One reason is that normative accounts do not distinguish between action and beliefs about action (control). These beliefs entail both content (expectations) and confidence (precision). This means that both expectations about behaviour and the precision of these beliefs have to be optimized. It is this optimization of precision that provides a complete account of bounded rationality (approximate Bayesian inference) and a plausible account of the control of dopaminergic firing (c.f. [64]).

Clearly, this account of dopamine does not address many important issues in the neurobiology of dopamine and its modelling. As with most free energy formulations, the objective is not to replace existing accounts but to contextualize them—usually by appealing to simpler and more fundamental imperatives. For example, we have seen that minimizing surprise (or its free energy bound) provides a principled account of goal-directed behaviour that is not biologically implausible. Furthermore, this account is consistent with many established formulations, converging on softmax choice rules, reconciling the contribution of intrinsic and extrinsic rewards and accounting for a range of anatomical and physiological properties of dopaminergic projections. Having said this, it remains an outstanding challenge to understand more detailed models of dopaminergic function in terms of approximate Bayesian inference. For example, several models consider tonic dopamine firing to influence action *per se* [65]. Others consider its effects on performance and learning [66]. Many studies suggest that the performance effects of dopamine can be explained in terms of costs and benefits—such that high dopamine levels allow an animal to ignore the costs of actions if the benefit is sufficiently high.

Action costs in variational (Bayesian) formulations are normally treated in terms of prior beliefs about control [47]—such that a costly action is unlikely *a priori*. A differential effect of dopamine on costs (prior beliefs about control states) and benefits (prior beliefs about hidden states) speaks to the possibility that these beliefs are equipped with their own precision—and the fact that dopaminergic systems have a multitude of neuromodulatory mechanisms (e.g. D1 versus D2 receptor targets, direct versus indirect pathways, nigrostriatal versus mesocortical projections, etc.). Perhaps the deeper question here is not about whether dopamine mediates expected precision but which beliefs or probabilistic representations are contextualized in terms of their precision. For example, pathophysiology involving the nigrostriatal system is likely to produce very different deficits when compared with abnormal mesocortical dopaminergic function. In short, the challenge may be to map the physiological and anatomical diversity of dopaminergic projections to the plurality of functions in which dopamine has been implicated [61,34,67]. Much progress has been made along these lines—and the encoding of precision may provide a common computational role for dopamine that is consistent with its (neuromodulatory) mechanism of action.

We have previously asserted that the values of *states* are the consequence of behaviour, not its cause [68]. The current formulation finesses this assertion because the value of an *action* is both cause and consequence of behaviour. This is self-evidently true by the circular causality implicit in the action perception cycle [69] of embodied (active) inference. This fits comfortably with the finding of action-value coding in the brain prior to overt choice—for both positive and negative action values [70,71]. Furthermore, optogenetic studies show that stimulating distinct populations of striatal neurons during choice can effectively add or subtract action-value and bias behaviour to select or avoid an associated action [72]. Stimulating these populations during outcome induces subsequent approach or avoidance [73]. These results again point to the plurality of postsynaptic dopaminergic effects. In this instance, converse effects on action selection depending upon whether (facilitatory) D1 receptors or (inhibitory) D2 receptors are activated. These complementary effects of dopaminergic innervation are potentially important in the context of encoding precision in hierarchical models that may underlie action selection [35]: in computational terms, a key determinant of posterior expectations is the relative precision at different levels of hierarchical representations. It may be the case that dopaminergic projections mediate the relative precision or confidence in representations [34]—in a way that relies upon the balanced opposition of distinct pathways or receptor subtypes [67].

In conclusion, the account on offer considers dopamine to report the precision of divergence or prediction errors (in their nuanced sense) and partly resolves the dialectic between dopamine as reporting reward prediction errors [29] and the predictability of rewards [59,60,74]. The notion that dopamine encodes precision is now receiving support from several lines of evidence from theoretical treatments of hierarchical Bayesian inference [63], theoretical neurobiology [31,35,32,34] and empirical studies [75–78]. Having said this, a proper validation of the active inference will require careful model comparison using empirical choice behaviours and a detailed mapping between putative model variables and their neuronal correlates. The approach adopted in this paper highlights the intimate relationship between inferring states of the world and optimal behaviour [79,80], the confidence or precision of that inference [81] and the functional plurality of dopaminergic neuromodulation [61].
